# Epigenetic characterization of sarcopenia-associated genes based on machine learning and network screening

**DOI:** 10.1186/s40001-023-01603-8

**Published:** 2024-01-16

**Authors:** Yong Chen, Zhenyu Zhang, Xiaolan Hu, Yang Zhang

**Affiliations:** 1 Key Laboratory of Renal Diseases Occurrence and Intervention of Hubei Province, Medical College, Hubei Polytechnic University, Huangshi, 435003 China; 2 Shenzhen Qihuang Guoyi Hanfang Innovation Research Center, Shenzhen, 518046 China; 3 Huangshi Central Hospital, Affiliated Hospital of Hubei Polytechnic University, Huangshi, 435099 China; 4 Pingshan District People’s Hospital of Shenzhen, Pingshan General Hospital of Southern Medical University, No. 19 Renmin Street, Pingshan Street, Pingshan District, Shenzhen, 518118 Guangdong China

**Keywords:** Sarcopenia, Machine learning, Bioinformatics, Epigenetics, Network screening

## Abstract

To screen characteristic genes related to sarcopenia by bioinformatics and machine learning, and to verify the accuracy of characteristic genes in the diagnosis of sarcopenia. Download myopia-related data sets from geo public database, find the differential genes through R language limma package after merging, STRING database to build protein interaction network, and do Go analysis and GSEA analysis to understand the functions and molecular signal pathways that may be affected by the differential genes. Further screen the characteristic genes through LASSO and SVM-RFE machine algorithms, make the ROC curve of the characteristic genes, and obtain the AUC value. 10 differential genes were obtained from the data set, including 7 upregulated genes and 3 downregulated genes. Eight characteristic genes were screened by a machine learning algorithm, and the AUC value of characteristic genes exceeded 0.7. In patients with sarcopenia, the expression of TPPP3, C1QA, LGR5, MYH8, and CDKN1A genes are upregulated, and the expression of SLC38A1, SERPINA5, and HOXB2 genes are downregulated. The above genes have high accuracy in the diagnosis of sarcopenia. The research results provide new ideas for the diagnosis and mechanism research of sarcopenia.

## Introduction

Sarcopenia is a syndrome of aging skeletal muscle mass reduction with decreased skeletal muscle strength and function [1], which can be classified as primary and secondary depending on the cause. Primary sarcopenia is mainly associated with age, secondary sarcopenia has obvious causes other than age, including poor exercise patterns, chronic wasting diseases, and impaired nutrient absorption [2]. In 2010, the European Working Group on Sarcopenia in the Elderly (EWGSOP) published a consensus on the definition and diagnosis of Sarcopenia, which has been recognized worldwide [3]. Sarcopenia is a relatively common disease in the elderly, and its prevalence increases with age. The survey found that the prevalence of over 65 years old is 5–13% and over 80 years old is 50% in the population of European countries [4]. In Asia, the prevalence rate in Japan was 7% for women aged 60–69 years 24% for men aged 70–80 years, 33% for men aged 60–69 years, and 47% for men aged 70–85 years [5]. A serious consequence of Sarcopenia is that it leads to a decrease in skeletal muscle strength, slowness, and balance in the elderly, making them prone to falls and fractures, greatly reducing the quality of life and increasing the burden on families and society. In 2015, China’s population aged 60 and above had reached 222 million, accounting for 16.1% of the total population. With the global trends in population aging, the number of people over 60 years old in China will increase and is expected to reach 400 million by 2050, accounting for about 25% of the total population. The quality of life of the elderly is related to the economic stability and development of our country and has attracted wide attention from the whole society.

Sarcopenia, first proposed by Evans WJ and Rosenberg IR in 1991, is a condition characterized by a reduction in bone-invasive muscle, but also involves mitochondrial dysfunction, impaired protein synthesis and degradation, autophagy and satellite cell activation, and other factors considered to be the underlying pathophysiological basis for sarcopenia. The hazards of sarcopenia mainly include increasing the risk of falls, fractures, and disabilities, making the elderly lose the ability to live independently, increasing the incidence of hypertension, diabetes, and other chronic diseases in the elderly, and increasing the all-cause mortality and disability rate of the elderly. Muscles and bones interact and correlate with each other. Some researchers have confirmed through systematic review that sarcopenia is significantly associated with the incidence of fracture, and male patients with sarcopenia and low bone mineral density have a significantly increased risk of fracture [6]. Decreased muscle function and reduced mass are risk factors for the development of falls [7, 8]. Therefore, the pathogenesis of sarcopenia needs to be studied, which provides a new target and theoretical reference for the future treatment of myopathy or skeletal sarcopenia associated with delaying aging.

Sarcopenia-osteoporosis has been used to describe the co-existence of sarcopenia and osteoporosis, both of which have a diminished motor system and loss of mass with age, focusing on the skeleton and muscle, respectively, and it has been claimed that "they are manifestations of one disease in different physiological systems [9]. There are no uniform criteria for the diagnosis of Sarcopenia in China and abroad. The European Working Group on Sarcopenia in the Elderly (EWGSOP), the Asian Working Group on Sarcopenia (AWGS), the International Working Group on Sarcopenia (IWGS), and the National Foundation for Sarcopenia Programs (FNIH) mainly diagnose Sarcopenia based on muscle mass, muscle strength, and muscle function. such as Table 1. The lack of characteristic genes and molecular markers and the unclear molecular mechanism make the treatment and prognosis of Sarcopenia difficult.

**Table 1:** The lack of characteristic genes and molecular markers and the unclear molecular mechanism makes the treatment and prognosis of Sarcopenia difficult

Research institute	Decreased muscle mass	Decreased muscle strength	Muscle hypofunction
EWGSOP	Less than healthy muscle mass Adult #2 standard deviations	Muscle strength is lower than healthy Adult #2 standard deviations	Pace lower than healthy Adult #2 standard deviations
AWGS	Limb muscle mass/height 2: Male < 7.0 kg/m^2^ Female < 5.4 kg/m^2^	Muscle strength: Male < 26 kg Female < 18 kg	Step speed < 0.8 m/s
IWGS	Extremity muscle mass/height^2^: Male ≤ 7.23 kg/m^2^ Female ≤ 5.67 kg/m^2^	–	Step speed < 1 m/s
FNIH	Extremity muscle mass/height^2^/BMI^*^: Male < 0.789 Female < 0.512	Muscle strength: Male < 26 kg Female < 16 kg	Step speed ≤ 0.8 m/s

^#^ Healthy adults: 19–39 years old; *BMI = body mass (kg)/height (m)^2^

The concept of epigenetics was first proposed by Waddinglon in 1942, who referred to epigenetics as the study of biological developmental mechanisms [10]. By the mid-1970s, R. Holliday considered epigenetics as the study of heritable changes in gene expression due to non-DNA sequence changes [11], which is now a more widely accepted concept. Epigenetic modifications play an important role in the development and progression of cancer and subsequent cachexia. Epigenetic modifications describe changes in the state of chromatin condensation and ultimately determine the accessibility of DNA to proteins that control transcription. Three major epigenetic mechanisms are said to play a key role in cancer development.

Bioinformatics is a new science that uses computer science and information technology to collect, process, analyze, and interpret a large number of bioinformatics data. Bioinformatics analysis technology is accompanied by the rapid development of life sciences and computer science, providing an efficient analysis tool for revealing the potential significance of large and complex biological data [12, 13]. Microarray microarray assay data from public data repositories such as GEO can then be screened for potentially relevant genes and related signaling pathways for disease prevention, diagnosis, and treatment using bioinformatics analysis methods. Many candidate genes for osteoporosis and sarcopenia have been identified and confirmed, but these studies still suffer from low reproducibility of the identified genes and lack of functional validation of the candidate genes. Therefore, we aimed to screen characteristic genes related to sarcopenia by bioinformatics and machine learning and to verify the accuracy of characteristic genes in the diagnosis of sarcopenia.

## Materials and methods

### Data collection and preprocess

We launched a search in the GEO database (https://www.ncbi.nlm.nih.gov/geo/) using “sarcopenia”, and “homo sapiens” as the keywords. Through searching and screening, we found that the samples of GSE1428 and GSE136344 met the conditions of the article, so they were included in this study. Among them, GSE1428 included 10 samples of young people and 12 samples of elderly people, and GSE136344 included 11 samples of young people and 12 samples of elderly people. To increase the sample size, the data from the two datasets were merged, and the combined data had 21 young and 24 old normal tissue samples. We combined the two gene expression profiling datasets using the ‘‘merge” function in the R package ‘‘base”. We adjusted for batch effects and normalized combined data using the ‘‘normalize Between Arrays” function in the R package ‘‘limma” [14].

### Differential expression analysis

The expression matrix consisting of young normal tissue samples and old normal tissue samples was obtained. The differentially expressed genes (DEGs) in young normal tissue samples compared with old normal tissue samples were then identified using the R Bioconductor limma package with the thresholds of *P*-values < 0.05, and |log2FC|≥ 1. At present, the absolute value of log2FC is generally used to represent the DEG threshold. The larger the value, the greater the difference between the two groups for the variable. The minimum value of log2FC is 1, indicating that the difference between the two groups for the variable is twice, indicating statistical significance. The heat map of DEGs was visualized with “heatmap” [15] and the volcano map was visualized with “ggplot2” [16].

### Gene ontology (GO) and GSEA analysis of DEGs

GO is commonly used to annotate gene function [17], mainly including the molecular function (MF), and cellular component (CC). To explore the role of DEGs in the mechanism of Sarcopenia, we used the R language clusterProfiler package [18] to perform GO analysis of DEGs. In addition, We choose c2.cp.kegg.v7.4.symbols.gmt geneset collection as the reference gene set, and perform GSEA analysis of DEGs [19]. to understand the relevant molecular signaling pathways that may be affected.

### Screening for signature genes

The least absolute shrinkage and selection operator (LASSO) logistic regression [20] with the “glmnet” package (version 4.1-1) and the support vector machine-recursive feature elimination (SVM-RFE) [21] with the “e1071” package (version 1.7-6) were applied to screen the specific genes. The obtained results of the two algorithms were intersected. LASSO is a generalized linear model that adds a regularization term to the ordinary least squares method, reducing the complexity and overfitting of the model by weighting the absolute value size on each feature. When using LASSO for logistic regression, it can effectively minimize the risk of the model while selecting features. LASSO can perform gene screening by fitting the LASSO model and selecting features with absolute coefficients greater than a certain threshold. SVM RFE is a feature selection method based on Support Vector Machine (SVM). It evaluates the importance of each feature by training an SVM classifier and retains the most important feature in the model. Then, this process is recursively applied to the remaining feature sets until a certain stopping condition is reached (such as the number of reserved categories). SVM-RFE can be sorted based on the degree of influence of features on classification performance, so it can perform gene screening in gene expression data. Both methods are based on the evaluation of feature importance or influence on feature selection. LASSO usually focuses more on determining the significance and physical significance of coefficients, while SVM-RFE focuses on determining the degree of influence of features on the final model performance in a certain sense.

### Protein–protein interaction (PPI) network analysis

To reveal the role of target proteins at the system level, the STRING database was used to import the mapped genes obtained from the previous step to construct the mapped target PPI, and the core targets were screened by setting the scores.

### Verification of gene signature

To evaluate the diagnostic accuracy of the signature genes, we conducted a ROC analysis of the signature genes, with a screening criterion of the area under the curve (AUC). When the ACU value > 0.7, it indicates that the characteristic gene is more accurate in the diagnosis of Sarcopenia. Receiver operating characteristic curve (ROC) analysis was conducted using the R package “pROC” [22], and the predictive performance was assessed by calculating the AUC.

### Statistical analysis

All statistical analyses were completed using R software. The DEGs were analyzed with the "limma" package, with the threshold set to |log2FC|> 1 and *P* < 0.05. The Wilcoxon non-parametric test was used to compare the two groups. A *P* < 0.05 was considered statistically significant.

## Results

### Identification of significant DEGs between old and young normal tissue samples

**Fig. 1:**
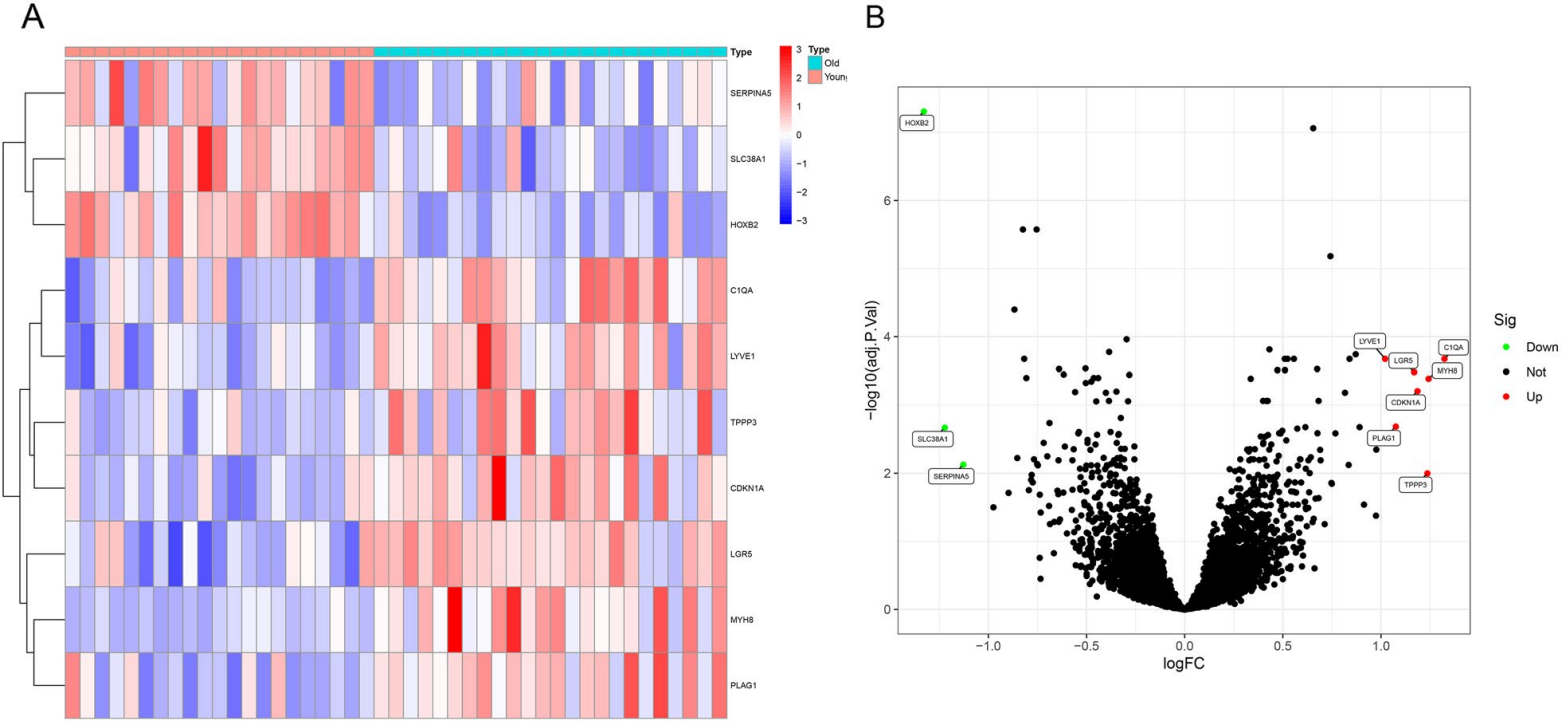
Identification of significant differentially expressed genes (DEGs) in Sarcopenia. **A** Heat map showing the DEGs identified. **B** Volcano plot showing the DEGs identified. Seven upregulated genes had a log2FC ≥ 1 and an adjusted *P*-value < 0.05. three downregulated genes had a log2FC ≤ − 1 and an adjusted *P*-value < 0.05

We cross-validate the selected genes using SVM-RFE to understand how many genes have the lowest error rate when extracted. The results show that when *n* = 10, the error rate of cross-validation is the lowest. Differential expression analysis identified 10 DEGs in old normal tissue samples compared with young normal tissue samples. including 7 upregulated genes (log2FC ≥ 1) and 3 downregulated genes (log2FC ≥ 1), as shown in the heat map and Volcano figure (Table 2 and Fig. 1).

**Table 2:** Identification of the DEGs in in old normal tissue compared with young normal tissue

Gene	logFC	AveExpr	*t*	*P*.val	adj.*P*.val
C1QA	1.323	5.725	5.919	< 0.01	< 0.01
MYH8	1.242	7.348	5.564	< 0.01	< 0.01
TPPP3	1.236	6.387	4.148	< 0.01	< 0.01
CDKN1A	1.186	6.409	5.416	< 0.01	< 0.01
LGR5	1.169	7.349	5.705	< 0.01	< 0.01
PLAG1	1.075	4.464	4.963	< 0.01	< 0.01
LYVE1	1.021	6.223	5.988	< 0.01	< 0.01
SERPINA5	− 1.128	7.012	− 4.305	< 0.01	< 0.01
SLC38A1	− 1.222	6.797	− 4.933	< 0.01	< 0.01
HOXB2	− 1.329	6.607	− 9.151	< 0.01	< 0.01

### Screening and verification of key genes

**Fig. 2:**
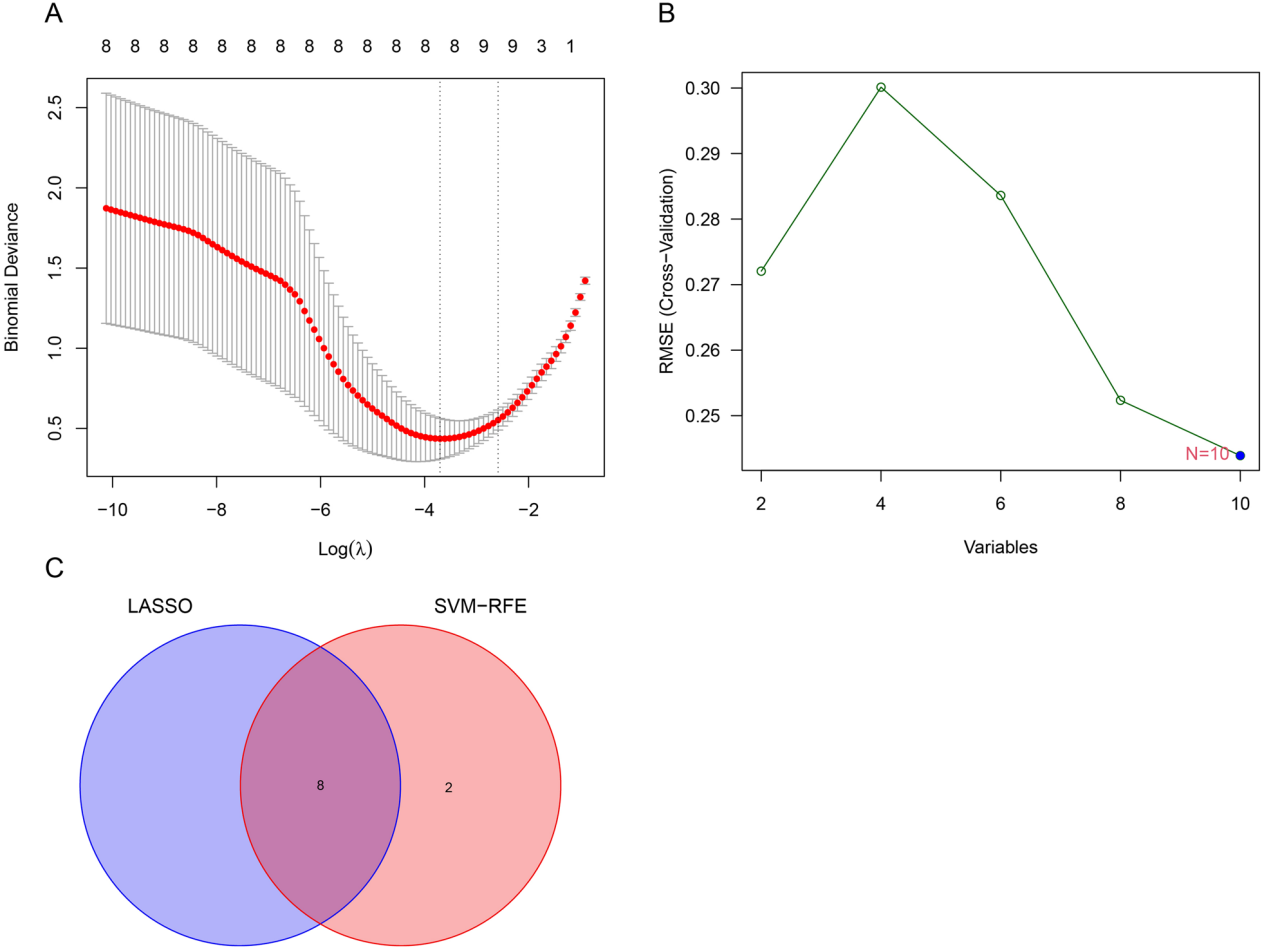
Screening of candidate genes. **A** LASSO logistic regression algorithm to screen candidate feature genes. **B** SVM-RFE algorithm to screen candidate genes. **C** The Venn diagram shows the intersection of genes obtained by two algorithms

To identify the potential genes with strong diagnostic significance value and biological significance from DEGs, we first used the LASSO regression algorithm and the SVM-RFE algorithm. we overlapped the genes identified by these three algorithms and finally obtained eight candidate genes: *C1QA*, *MYH8*, *TPPP3*, *CDKN1A*, *LGR5*, *SEPRINA5*, *SLC38A1*, and *HOXB2* (Fig. 2).

### Validation and efficacy evaluation of signature genes

**Fig. 3:**
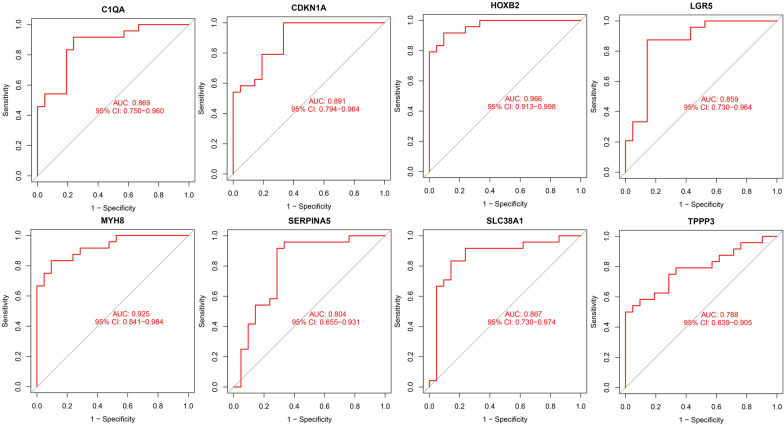
ROC curve of signature genes including C1QA, MYH8, TPPP3, CDKN1A, LGR5, SERPINA5, SLC38A1, and HOXB2

ROC curve was plotted and the area under the curve (AUC) was calculated to distinguish the old normal tissue samples from the young normal tissue samples, and every AUC of the eight candidate genes was greater than 0.7 in our study. The ROC curve showed that eight candidate genes had a good diagnostic efficiency (both AUC > 0.844, Fig. 3).

### PPI network analysis

**Fig. 4:**
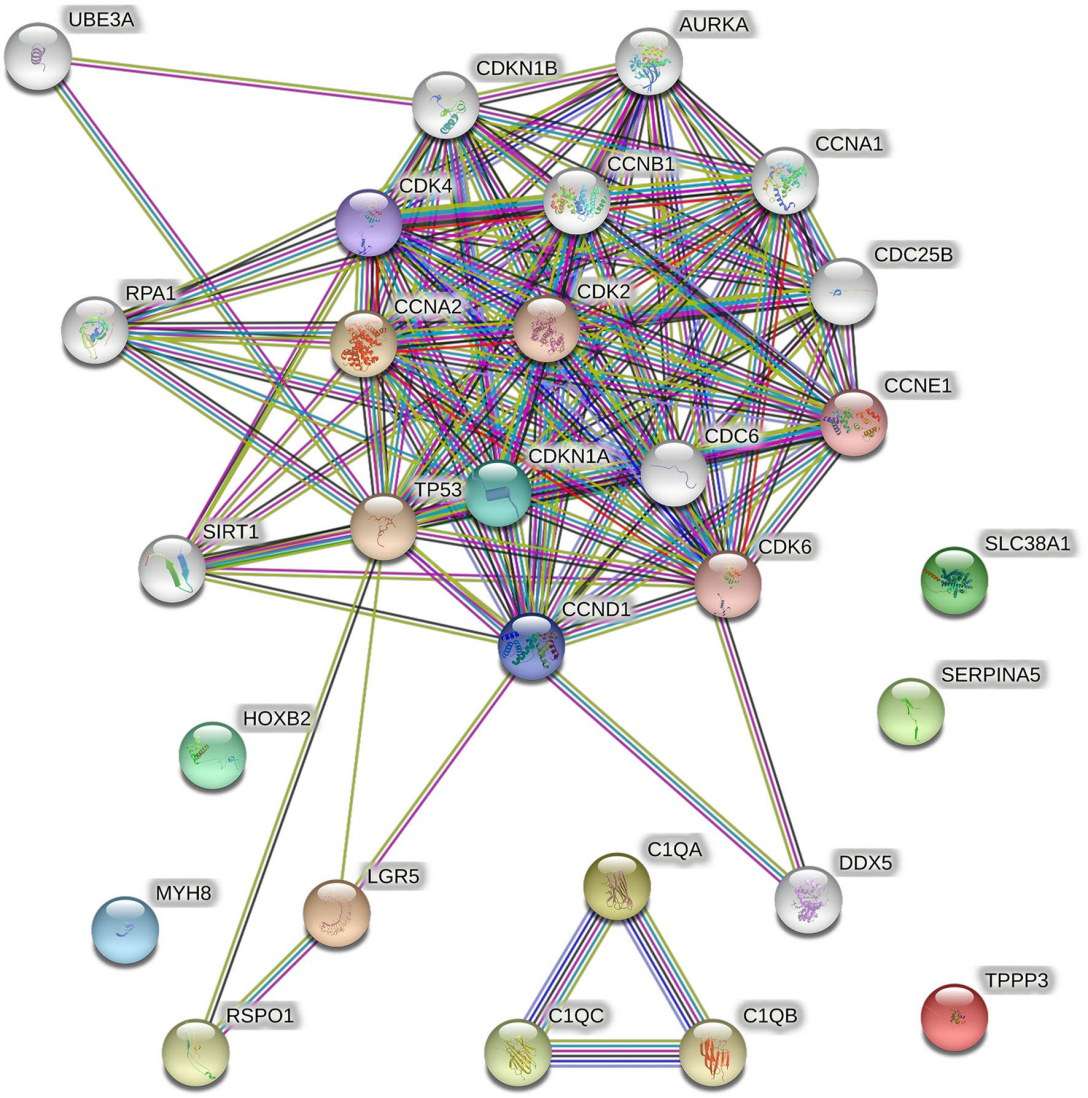
PPI Network plot of differentially expressed genes (drawn by the STRING database 11.0, https://string-db.org/) A PPI network complex with 28 nodes and 120 edges

Through PPI analysis, other proteins with significant expression difference multiples were also analyzed in this study to screen out some proteins of interest that may be clinically important, as shown in Fig. 4, where the larger the Degree value of the nodes, the darker the color and the larger the diameter. The possible mechanism of their expression in Sarcopenia and their potential as candidate markers were tentatively explored.

### GO pathway enrichment analysis

To predict the underlying biological function and corresponding pathways of these significant DEGs, the DAVID database was introduced to perform functional enrichment analysis, including two GO terms (CC: cellular component; MF: molecular function).

**Fig. 5:**
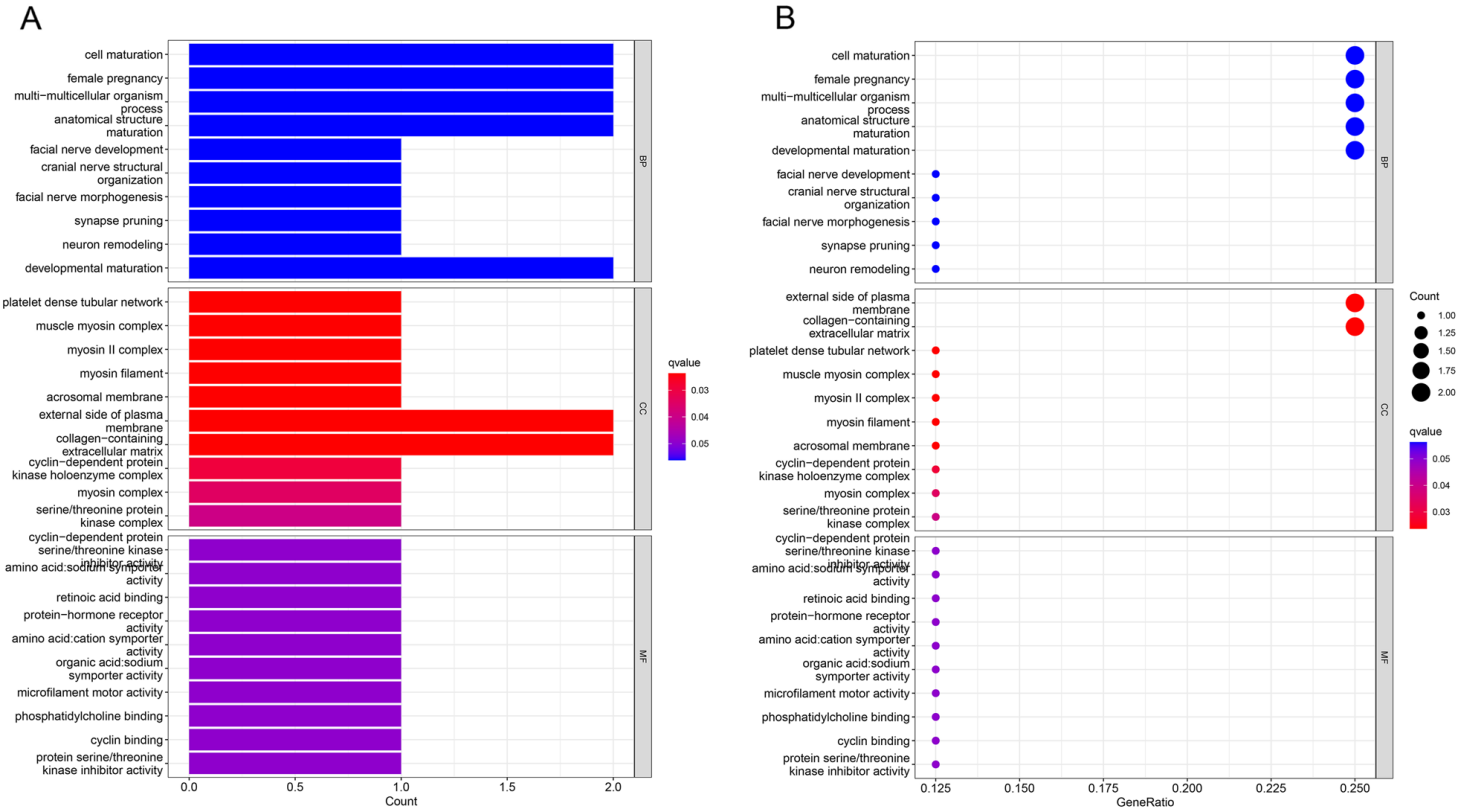
GO functional annotation for the significant DEGs. **A** The horizontal axis represents the number of DEGs under the GO term. **B** The air bubble diagram represents the number of DEGs under the GO term

For upregulated significant DEGs, as presented in Table 3 and Fig. 5A and B, the enriched GO functions included platelet dense tubular network, muscle myosin complex, myosin II complex, and myosin filament in the CC category; and cyclin-dependent protein serine/threonine kinase inhibitor activity, amino acid: sodium symporter activity, retinoic acid binding, protein-hormone receptor activity and amino acid: cation symporter activity in the MF category.

**Table 3:** GO Enrichment Analysis of the DEGs

ONTOLOGY	ID	Description	*P*-value
CC	GO:0031094	Platelet dense tubular network	< 0.01
MF	GO:0004861	Cyclin-dependent protein serine/threonine kinase inhibitor activity	< 0.01
CC	GO:0005859	Muscle myosin complex	< 0.01
MF	GO:0005283	Amino acid: sodium symporter activity	< 0.01
CC	GO:0016460	Myosin II complex	< 0.01
MF	GO:0001972	Retinoic acid binding	< 0.01
MF	GO:0016500	Protein-hormone receptor activity	< 0.01
CC	GO:0032982	Myosin filament	< 0.01
MF	GO:0005416	Amino acid: cation symporter activity	< 0.01

*CC* Cellular component, *MF* molecular function, *DEGs* differentially expressed genes, *GO* Gene Ontology

### GSEA analysis

**Fig. 6:**
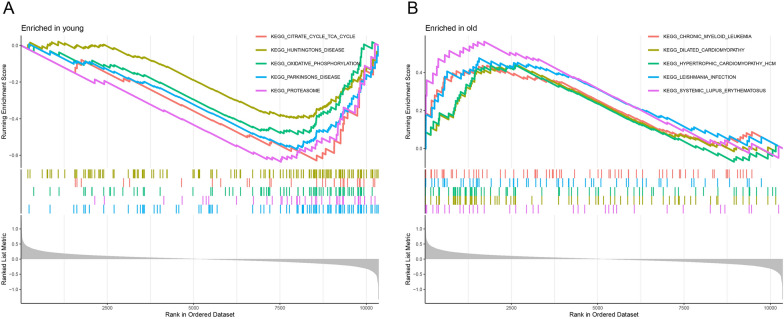
Gene set enrichment analysis. **A** Gene set enrichment analysis illustrated upregulated gene sets in the young normal tissue samples. **B** Gene set enrichment analysis illustrated upregulated gene sets in the old normal tissue samples

To investigate the biological processes associated with HCC early recurrence, gene set enrichment analysis (GSEA) was performed with hallmark pathways based on the gene expression profiling data from Sarcopenia patients in the young and old normal tissue samples. Many signaling pathways were significantly enriched in the old normal tissue samples, such as Cardiovascular disease-related pathways, Neurological disease-related pathways, and Metabolism-related pathways, while no significant gene set enrichment was observed in the young normal tissue samples (Fig. 6, Table 4).

**Table 4:** GSEA pathways upregulated in the old normal tissue samples

ID	Enrichment score	NES	*P* values
KEGG_SYSTEMIC_LUPUS_ERYTHEMATOSUS	0.563	1.841	< 0.01
KEGG_LEISHMANIA_INFECTION	0.476	1.705	< 0.01
KEGG_DILATED_CARDIOMYOPATHY	0.444	1.694	< 0.01
KEGG_HYPERTROPHIC_CARDIOMYOPATHY_HCM	0.438	1.639	< 0.01
KEGG_CHRONIC_MYELOID_LEUKEMIA	0.437	1.641	< 0.01
KEGG_HUNTINGTONS_DISEASE	− 0.397	− 1.715	< 0.01
KEGG_OXIDATIVE_PHOSPHORYLATION	− 0.486	− 1.923	< 0.01
KEGG_PARKINSONS_DISEASE	− 0.567	− 2.277	< 0.01
KEGG_CITRATE_CYCLE_TCA_CYCLE	− 0.629	− 2.036	< 0.01
KEGG_PROTEASOME	− 0.636	− 2.184	< 0.01

## Discussion

The global population is aging at an accelerating rate, and with age comes changes in the composition of the body, including fatty tissue and muscle. Skeletal muscle decreases gradually after age 50 and muscle mass and strength also decreases gradually. Sarcopenia is a syndrome of progressive, widespread loss of skeletal muscle mass and strength, and the resulting adverse consequences of decreased physical performance, decreased quality of life, and death [3]. As more research emerges, the most widely cited definition currently comes from the EWGSOP2 consensus proposed by the 2018 EWGSOP, which identified decreased muscle strength as the primary parameter for the assessment of sarcopenia, arguing that sarcopenia may be present when it is found sarcopenia is diagnosed when low muscle quantity or quality is found, and severe sarcopenia is when low muscle strength, low muscle quantity or quality, and physical function are all present [23]. Sarcopenia is a common disease among the elderly, and its incidence is increasing with age. However, at present, its diagnosis can only be made by combining the indicators of muscle volume, muscle strength, and muscle function, lacking specific molecular markers, and its molecular mechanism is also complicated. Various pathological factors, including oxidative stress, inflammatory response, and insulin resistance, are known to be involved in the formation of Sarcopenia, which ultimately leads to decreased protein synthesis and increased catabolism in skeletal muscle cells and the conversion of muscle fibers from type II fibers (fast muscle fibers) to type I fibers (slow muscle fibers) [24].

In this study, we found that *TPPP3*, *C1QA*, *LGR5*, *MYH8*, *CDKN1A* gene expression was upregulated, while *SLC38A1*, SERPINA5, *HOXB2* gene expression was downregulated and the AUC value was greater than 0.7 in elderly muscles compared to young people, suggesting that these genes and their encoded proteins have the potential to be diagnostic markers for sarcopenia. Among them, *MYH8*, HOXB2, *C1QA*, *CDKN1A,* and *SLC38A1* are associated with sarcopenia [25], and in this study, *LGR5*, *SERPINA5,* and *TPPP3* genes were found to be associated with Sarcopenia for the first time. the protein encoded by *LGR5* is a newly discovered G protein-coupled receptor in recent years and is involved in the classical Wnt signaling pathway [26], Interestingly, the Wnt signaling pathway is involved in skeletal muscle production and development [27]. *SERPINA5* encodes a protein that is a glycoprotein that inhibits a variety of serine proteases, including protein C, various fibrinogen activators, and kinin-release enzymes [28]. *TPPP3* encodes a pro-microtubule polymerization protein that specifically binds to microtubules in vitro and in vivo and may play a role in pro-microtubule aggregation into bundles, cell proliferation, and mitosis [29]. All of these genes encode proteins that are associated with muscle composition and function and are likely to play a role in the development of Sarcopenia.

The present study also has some limitations, such as a small sample size to better set up biological replicates, and it is only a theoretical study under bioinformatics analysis; the next step should be to perform some molecular biology experiments for validation, to further elucidate the specific mechanisms and effects of sarcopenia activation in vitro.

This study analyzed the possibility of differential genes as a diagnostic molecule for Sarcopenia and explored its possible molecular mechanisms, providing new ideas for the diagnosis of Sarcopenia and the exploration of molecular mechanisms.

## Conclusions

In conclusion, our results suggest that the expression of TPP3, C1QA, LGR5, MYH8, and CDKN1A genes are upregulated in sarcopenic patients, while the expression of SLC38A1, SERPINA5, and HOXB2 genes are downregulated and can be used as biomarkers for the diagnosis of sarcopenic patients.

## Data Availability

The datasets analyzed during the current study are available in the TCGA repository, https://www.ncbi.nlm.nih.gov/geo/.
